# Slow Waves Promote Sleep-Dependent Plasticity and Functional Recovery after Stroke

**DOI:** 10.1523/JNEUROSCI.0373-20.2020

**Published:** 2020-11-04

**Authors:** Laura Facchin, Cornelia Schöne, Armand Mensen, Mojtaba Bandarabadi, Federica Pilotto, Smita Saxena, Paul Antoine Libourel, Claudio L.A. Bassetti, Antoine R. Adamantidis

**Affiliations:** ^1^Centre for Experimental Neurology, Department of Neurology, Inselspital University Hospital, University of Bern, 3010, Bern, Switzerland; ^2^Department of Neurology, Inselspital University Hospital, University of Bern, Bern, 3010, Switzerland; ^3^Department for BioMedical Research, University of Bern, Bern, 3010, Switzerland; ^4^Centre de Recherche en Neurosciences de Lyon, University of Lyon, Bron, 69500, France

**Keywords:** ischemic stroke, neuroplasticity, optogenetics, slow wave sleep

## Abstract

Functional recovery after stroke is associated with a remapping of neural circuits. This reorganization is often associated with low-frequency, high-amplitude oscillations in the peri-infarct zone in both rodents and humans. These oscillations are reminiscent of sleep slow waves (SW) and suggestive of a role for sleep in brain plasticity that occur during stroke recovery; however, direct evidence is missing. Using a stroke model in male mice, we showed that stroke was followed by a transient increase in NREM sleep accompanied by reduced amplitude and slope of ipsilateral NREM sleep SW. We next used 5 ms optical activation of Channelrhodopsin 2-expressing pyramidal neurons, or 200 ms silencing of Archeorhodopsin T-expressing pyramidal neurons, to generate local cortical UP, or DOWN, states, respectively, both sharing similarities with spontaneous NREM SW in freely moving mice. Importantly, we found that single optogenetically evoked SW (SW^opto^) in the peri-infarct zone, randomly distributed during sleep, significantly improved fine motor movements of the limb corresponding to the sensorimotor stroke lesion site compared with spontaneous recovery and control conditions, while motor strength remained unchanged. In contrast, SW^opto^ during wakefulness had no effect. Furthermore, chronic SW^opto^ during sleep were associated with local axonal sprouting as revealed by the increase of anatomic presynaptic and postsynaptic markers in the peri-infarct zone and corresponding contralesional areas to cortical circuit reorganization during stroke recovery. These results support a role for sleep SW in cortical circuit plasticity and sensorimotor recovery after stroke and provide a clinically relevant framework for rehabilitation strategies using neuromodulation during sleep.

**SIGNIFICANCE STATEMENT** Brain stroke is one of the leading causes of death and major disabilities in the elderly worldwide. A better understanding of the pathophysiological mechanisms underlying spontaneous brain plasticity after stroke, together with an optimization of rehabilitative strategies, are essential to improve stroke treatments. Here, we investigate the role of optogenetically induced sleep slow waves in an animal model of ischemic stroke and identify sleep as a window for poststroke intervention that promotes neuroplasticity and facilitates sensorimotor recovery.

## Introduction

Stroke is an acute brain injury caused by a sudden decrease in cerebral blood flow, followed by local inflammation (Huang et al., 2006), excitotoxicity (Lai et al., 2014), and cell death (Small et al., 1999). Changes in neuronal excitability after stroke are thought to promote long-term plasticity in surviving neurons that contributes to the reorganization of cortical maps and to the underlying level of axonal sprouting supporting brain functions (Carmichael, 2012; van Meer et al., 2012; Silasi and Murphy, 2014), as observed in rodents (Nudo, 1997; Murphy and Corbett, 2009; Carmichael et al., 2017) and humans (Khedr et al., 2005; Lindenberg et al., 2010). To date, pharmacological treatments and noninvasive brain neuromodulation techniques hold promise in improving plasticity and functional recovery both in animal models (Zhang et al., 2007; Yoon et al., 2012) and in humans (Robinson et al., 2008; Ameli et al., 2009; Talelli et al., 2012), yet the underlying mechanisms remain uncler.

Poststroke hyperexcitability of surviving neurons contributes to the transient low-frequency (∼1 Hz, 200-500 ms in duration), high-amplitude, rhythmic waves (also coined “bistable state”), originating in the peri-infarct zone and propagating to contralesional brain areas. This distinctive 1 Hz slow and synchronous neural activity in the peri-infarct zone shares similarities with slow waves (SWs) typically recorded during non-rapid eye movement (NREM) sleep in rodents and humans. Indeed, SWs reflect bistable states of thalamocortical neuron populations, described as a switch between UP states, where depolarized membrane potentials are accompanied by high spiking activity, and DOWN states during which cells are hyperpolarized, and show low spiking activities in cats (Steriade et al., 1993), rodents (Vyazovskiy et al., 2009a,b; Zucca et al., 2017), nonhuman primates (Xu et al., 2019), and humans (Csercsa et al., 2010). These SWs were hypothesized to guide axonal sprouting and circuit rewiring through the formation of new connections after brain lesions (Carmichael and Chesselet, 2002) facilitating recovery; however, this has not been directly demonstrated.

Extensive experimental evidence suggests a fundamental role for intact sleep, and SW in particular, in enhancing brain plasticity during spontaneous sleep (Timofeev and Chauvette, 2017; Tononi and Cirelli, 2020) and stroke recovery (Duss et al., 2017). The detrimental effects of sleep disturbances (Kaneko et al., 2003; Baglioni et al., 2016) and the beneficial effect of pharmacological NREM sleep enhancement after stroke support the hypothesis that SWs contribute to brain plasticity underlying poststroke functional and cognitive recovery both in animal models (Gao et al., 2008; Hodor et al., 2014) and patients (Vock et al., 2002; Siccoli et al., 2008; Sarasso et al., 2014).

Here, we used an optogenetic approach inspired by global and local SW changes after stroke to rescue SW-like activity in freely moving mice. Optogenetic activation of pyramidal neurons in the peri-infarct zone during NREM sleep improved fine motor movements compared with experimental control conditions. In contrast, optogenetically evoked SW (SW^opto^) during wakefulness had no effect. Importantly, SW^opto^ evoked recovery after stroke was associated with axonal sprouting in the peri-infarct zone and corresponding contralesional areas.

## Materials and Methods

### 

#### Animals

C57BL/6JRj male mice (https://www.janvier-labs.com/en/fiche_produit/c57bl-6jrj_mouse/; 5-6 weeks old, 23-30 g) were used in the study. Animals were individually housed in custom-designed polycarbonate cages (300 mm × 170 mm) under controlled conditions (regular circadian cycle of 12:12 h light:dark; light on at either 4:00 A.M. or 8:00 P.M. according to experimental design; constant temperature 22 ± 1°C and humidity 30%-50%). Throughout the experiment, animals were freely moving with *ad libitum* food and water. Animals were kept in groups of 2-5 per IVC cage before instrumentation and after viral injection surgery. Following implantation, mice were all housed individually. Animals were tethered, allowed to adapt to the EEG/EMG and optic stimulation cables in their home cage for at least 5-7 d, and remained plugged for the duration of the experiment. Animals were detached from all tethers for 4 d following stroke or sham surgery and for the duration of behavioral testing. Animals were randomly assigned to eight experimental groups: Channelrhodopsin (ChR2)-transfected animals subjected to stroke (ChR2^stroke^), ChR2-transfected animals subjected to stroke and optogenetically stimulated mainly during wakefulness (ChR2^stroke_wake^), Archaerhodopsin (ArchT)-transfected animals subjected to stroke (ArchT^stroke^), mCherry-transfected animals subjected to stroke (mCherry^stroke^), mCherry-transfected animals subjected to sham surgery (mCherry^sham^), Naive, Sham, and Stroke. Animals that displayed baseline asymmetry in limb usage or did not show a drop in cerebral blood flow by ∼80% during middle cerebral artery occlusion (MCAo) surgery were excluded from further experimental tests. Viral injections were performed when animals were 5-6 weeks of age, instrumentation at 8 weeks of age, and stroke/sham surgery at 10 weeks of age. Between surgeries and before being tethered, animals were allowed to recover undisturbed for at least 7 d. Naive mice did not undergo any surgical procedures. An additional set of heterozygous *Tg(VGAT-Cre)* mice (5-6 weeks old, 23-30 g) was used for an optogenetic screening of SW-like oscillations inducing protocols. All animals were treated according to animal care laws, and experimental procedures were approved by local authorities (Veterinary Office, Canton of Bern, Switzerland; license numbers BE 113/13 and BE 41/17).

#### Viral targeting

For a detailed description of the surgical procedure, refer to Herrera et al. (2016). Briefly, 5- to 6-week-old animals were anesthetized with isoflurane (4.0% induction; 1.0%-1.5% maintenance). Body temperature was constantly monitored and kept at physiological range using a rectal thermoprobe and feedback-controlled heating system. Animals were fixed in a digital stereotaxic frame, and analgesia was administered subcutaneously (meloxicam, 5 mg/kg). Animals were randomly assigned to receive 0.6 µl of recombinant AAV carrying CaMKII-hChR2 (H134)-EYFP (activation), CaMKIIa-eArchT3.0-EYFP (silencing), or CaMKIIa-mCherry (control), respectively. Plasmids were stereotactically injected (0.1 µl/min infusion rate) through a 28 G needle (Plastic One), connected by a tubing to a 10 µl Hamilton syringe in an infusion pump (model 1200, Harvard Apparatus). Injections were performed within the left (prospective ipsilateral) primary somatosensory forelimb cortex (iS1FL, AP: −0.10 mm; ML: −2.00 mm; DV: −0.7 mm). Animals were given 7 d of recovery before instrumentation surgery. *Tg(VGAT:Cre)* mice underwent identical surgical procedures as WT animals, randomly assigned to receive 0.6 µl of recombinant AVV carrying Ef1α-DIO-ChR2-EYFP (activation), Ef1α-DIO-ArchT-EYFP (silencing), or Ef1α-DIO-EYFP (control), respectively. All plasmids were obtained from the University of North Carolina Vector Core Facility. Mice belonging to Sham, Stroke and Naive groups did not received any AAV injection.

#### Instrumentation

Animals were chronically implanted with a unilateral optic fiber (200 µm in diameter) within the iS1FL (AP: −0.10 mm; ML: −2.00 mm; DV: −0.5 mm) and an EEG/EMG connector. As previously reported (Gent et al., 2018), animals received analgesia (meloxicam, 5 mg/kg), were anesthetized with isoflurane, and anchored to a stereotaxic frame. Five stainless-steel EEG electrode screws were inserted through each animal's skull: two screws over the frontal cortices (AP: 2 mm; ML: ±2 mm), two screws over the posterior cortices (AP: −4 mm; ML: ±2 mm), and one screw over the olfactory bulb as ground. For the stimulation recordings, the EEG signals from the frontal and posterior channels were referenced to each other directly, leaving only two EEG traces, one per hemisphere. Finally, two bare-ended EMG wires were sutured to the neck muscles to record postural tone. A subset of animals was additionally implanted with four tetrodes to record local field potentials (LFPs) and single-unit activity during optogenetic stimulation, as well as EEG/EMG signals. Tetrodes were constructed by twisting four tungsten wires together (10 µm in diameter, CFW0010954, California Fine Wire) and briefly heating them to favor the bond coating of each wire to another. Tetrodes were lowered within the iS1FL (AP: −0.10 mm; ML: −2.00 mm; DV: −0.5 mm), the ipsilateral primary motor cortex (iM1, AP: 1.10 mm; ML: −1.5 mm; DV: −1.20 mm), the contralateral S1FL (cS1FL, AP: −0.10 mm; ML: 2.00 mm; DV: −0.5 mm), and the contralateral M1 (cM1, AP: 1.10 mm; ML: 1.5 mm; DV: −1.20 mm) respectively. The tetrode positioned in iS1FL was glued to the optic fiber, where the tip of the tetrode extended for ∼0.2 mm beyond the end of the fiber (optrode). Optic fibers and implants were permanently secured to the skull with C&B Metabond (Patterson Dental) and methacrylate cement (Paladur). Animals were monitored postoperatively and left to recover undisturbed for at least 7 d. Animals were then plugged to the EEG/EMG/optic stimulation and tetrode tethers (Neuralynx headstage). Black nail polish was applied at the connection point between optic fiber and patch cord to limit laser light spreading during optogenetic stimulations. The implantation procedure for animals belonging to Sham and Stroke groups did not include either optic fiber or tetrode placement.

#### Transient focal cerebral ischemic stroke

Mice underwent MCAo via intraluminal filament model (Doeppner et al., 2010) at ∼10 weeks of age. To begin, mice were anesthetized with isoflurane as previously described and placed in a prone position. Physiologic temperature was maintained as mentioned above. The left common carotid artery (CCA) was dissected from the surrounding connective tissue. A monofilament suture (7-0 silicon rubber-coated, coating length 5-6 mm, Doccol) was inserted in the CCA and introduced into the lumen of the MCA. The monofilament was left in place for 45 min to induce both striatal and cortical infarct and consequently withdrawn to allow the reperfusion of the territory targeted by the MCA. Cerebral blood flow was constantly monitored by a Laser Doppler probe (Moor Instrument, VMS-LDF2) glued to the skull above the MCA region. Ischemic stroke induction was considered successful when the cerebral blood flow showed an ∼80% reduction from baseline values, as well as reperfusion of the MCA territory. Following surgery, mice were daily checked for pain and weight loss, received mashed, watered food, subcutaneous analgesia, and 0.9% saline. Animals belonging to the Naive group did not undergo stroke or sham surgery. No filament was inserted into the MCA during sham surgery. Following MCAo, 40% of animals assigned to the Stroke group and 33% of all animals allocated to optogenetic stimulations did not survive the postoperation phase.

#### Optogenetic stimulation

Lasers (Laserglow Technologies) attached to the unilateral fiber via patch cord (Thorlabs) were triggered through TTL with a pulse stimulator (Master-9, AMPI), this latter controlled by a function generator (Agilent Technologies, 33220A 20 MHz Function/Arbitrary waveform Generator) to induce random pulse sequences. Animals received daily 2 h of randomly distributed single laser light pulses (interpulses interval 3-30 s), from poststroke day 5 until day 15. The random distribution of light pulses was selected to avoid hypersynchrony and entrainment of oscillatory activities which, per se, might influence the observed parameters. The optogenetic stimulation was semichronic: light pulses were distributed across sleep and wake states without simultaneous behavioral scoring by the experimenter and consequent state specific stimulation. Indeed, daily and chronic stimulation (11 d) of several animals (experimental and control were run in parallel) is not suited for a single experimenter. The specific time allocated for optogenetic intervention was therefore selected according to the natural distribution of the majority of NREM sleep and wakefulness episodes throughout the 12 h light:dark cycle of the animals. Two stimulation protocols were used: ChR2-expressing animals received 5 ms blue light pulses (473 nm wavelength), ArchT-expressing mice were stimulated with 200 ms green light pulses (532 nm wavelength), and mCherry-expressing animals were randomly subjected to either 200 or 5 ms light pulses. To assess whether the effect of SW^opto^ on functional recovery was specific to brain activity occurring during sleep, in a separate group of animals (ChR2^stroke_wake^), optogenetic stimulations were also delivered during the first part of the dark phase, when animals were mostly awake. Based on pre-instrumentation testing of both optic fiber and patch cord outputs, light power was set at 20-25 mW.

#### Data acquisition

EEG and EMG signals were amplified (model 3500, AM System) and digitized at 512 Hz (NIDAQ 6363, National Instruments) using a sleep recording software (MATLAB written software, DaqReverse). A 24 h baseline of spontaneous sleep-wake behavior was recorded for all animals. Stroke and Sham animals were recorded for 24 h at postsurgery days 1, 3, 5, and 10. All optogenetic stimulations took place between 9:00 A.M. and 2:00 P.M., with light on at 4:00 A.M. for ChR2^stroke^, ArchT^stroke^, mCherry^stroke^, and mCherry^sham^. Since ChR2^stroke^ and ArchT^stroke^ animals showed similar functional outcomes on neuronal manipulation during sleep, an additional ChR2-transfected set of animals received SW^opto^ during animals' active phase (between 9:00 A.M. and 2:00 P.M., lights on at 8:00 P.M., ChR2^stroke_wake^), from poststroke day 5 until day 15. Animals' spontaneous sleep was recorded for 18 h at poststroke day 5, 6, 8, 12, and 14, respectively. LFPs and EEG/EMG signals were amplified and digitized at 32 kHz (Cheetah 5 acquisition software, Neuralynx; https://neuralynx.com/software/cheetah-5.0-legacy).

#### Behavioral tests

All animals were trained in four behavioral tests and engaged in daily training sessions for 3 consecutive days. Behavioral baselines were acquired before stroke/sham surgery. Functional outcomes were verified at poststroke days 4, 7, 10, and 15. All behavioral tests were conducted at least 3 h apart from optogenetic stimulations and during animals' active phase (between 5:00 P.M. and 8:00 P.M.). Test sessions were recorded with a picamera (Raspberry Pi) and scored in slow motion (VideoPad software; https://www.nchsoftware.com/videopad/index.html).

##### Balance beam test

To assess motor balance and coordination (Brooks and Dunnett, 2009), a round wooden beam (12 mm in diameter, 80 cm long) was positioned at an angle so that one end of the beam was 60 cm elevated from the working table. At the beam's elevated end, the animal's home cage served as motivation to complete the task. Soft fabric placed beneath the beam avoided possible falling injuries. The number of “paw faults” (forelimb or hindlimb slipping off the beam) were counted during a maximal testing time of 60 s. Each animal underwent three trials per time point and means were calculated.

##### Tight rope test

To measure grip strength and endurance (Balkaya et al., 2013), animals were suspended on a fine rope (60 cm above the working table) between two platforms (80 cm apart from one another). Mice were positioned at the middle point of the rope exclusively with their forepaws. The average time needed to reach one of the two platforms was calculated between two trials. The maximum testing time was 60 s.

##### Corner turn test

To evaluate the presence of unilateral abnormalities (Park et al., 2014), mice where placed in between two vertical boards forming a 30° angle. Animals' left- or right-turn decision was recorded for a total of 10 trials per testing session. Laterality index was calculated as (number of left turns – number of right turns)/10.

##### Ladder walking rig test

The test was chosen to measure paw accurate placement (Cummings et al., 2007). The apparatus consisted of a ladder (80 cm long), suspended between two platforms (60 cm above the working table) with randomly spaced rungs. Paw faults were recorded as animals walked to reach the home cage at one end of the ladder. Mice performances were scored in slow motion and the mean of three trials calculated. The position of the rungs was randomly changed across trials to avoid learning.

#### Signal processing

As previously described (Jego et al., 2013), electrophysiological data were manually scored in 5 s epochs and analyzed using SlipAnalysis (custom-written MATLAB program). Briefly, three vigilance states were identified based on EEG/EMG frequency and amplitude. Wakefulness was determined by low-amplitude EEG and high-activity EMG signals; NREM sleep as high-amplitude and low-frequency EEG (0.5-4 Hz) paired with reduced EMG activity; REM was characterized by theta rhythm (6-9 Hz) EEG and flat EMG. Microarousals were defined and scored as cortical fast rhythm and EMG bursts of at least 1 s. Sleep/wakefulness scoring was based on the visual characteristics of the contralateral EEG traces specifically. Electrophysiological analysis was completed using custom MATLAB scripts.

#### Automatic single SW detection

Individual SWs were detected during NREM sleep epochs during the first 7 h of the lights ON period in MATLAB using the SWA-MATLAB toolbox (Mensen et al., 2016), with detection parameters adjusted to rodents from settings described by Panagiotou et al. (2017). Briefly, in a first pass of the data, the negative envelope across the four EEG channels was calculated, filtered between 0.5 and 4 Hz (Chebyshev Type II filter design), and consecutive zero-crossings were detected. If the duration between successive downward (negative going) zero-crossing and upward zero-crossing was between 100 ms and 1 s, then the peak negative amplitude was examined and was required to be at least 3 deviations from the median amplitude of all negative peaks in the recording. The amplitude threshold eliminates the potential individual differences of electrode reference type, distance to those references, and electrode depth that would affect the record amplitude. In a second pass, the activity over all four channels was examined for each SW detected on the negative envelope to obtain individual channel data.

#### Single-unit analysis

We performed spike detection and sorting as described previously (Gent et al., 2018). Briefly, we first extracted multiunit activity from bandpass filtered signals (600-4000 Hz, fourth-order elliptic filter, 0.1 dB passband ripple, −40 dB stopband attenuation), by applying a detection threshold of 7.5 × the median of the absolute values of the filtered signal. We then extracted wavelet coefficients from the detected multiunit activity using a four-level discrete wavelet transform (Harr wavelet, “wavedec,” MATLAB), and subsequently sorted the coefficients using the superparamagnetic clustering. We visually inspected the sorted units and excluded the clusters with a symmetric shape or an average firing rate <0.2 Hz from our analyses.

#### Optogenetic response analysis

We assessed the optogenetic response analysis for each vigilance state separately. For unit activity, we calculated mean firing rates during optogenetic perturbations by averaging firing rates across trials using a nonoverlapping moving window of 5 ms. For LFP analysis, we averaged raw LFP signals across trials of each vigilance state.

#### Infarct volume evaluation and immunohistochemistry

Animals were killed at poststroke day 15 with 15 mg pentobarbital intraperitoneal injection (Esconarkon ad us. vet., Streuli Pharma) and transcardially perfused with 1× PBS followed by 4% formalin. Brains were postfixed overnight, cryoprotected in 30% sucrose (24-48 h at 4°C), frozen in 2-methyl-butane on dry ice and cut into 40 µm sections. Every third slice was mounted onto a glass slide, dried at room temperature, rehydrated, and processed for Nissl staining. Briefly, sections were immersed in Cresyl Violet (Klüver Barrera, Bio-Optica), washed in distilled water and dehydrated in graded alcohols, cleared in xylene (Sigma Millipore), and mounted (Eukitt mounting medium, Bio-Optica) on microscope slides. Stroke edges were delineated per section using ImageJ software (https://imagej.nih.gov/ij/). The damaged area was measured in each brain slice and multiplied by the distance between brain sections. Stroke volume relative to the whole brain was calculated as follows: ((volume of contralesional hemisphere – volume of ipsilesional hemisphere)/2 × volume of contralesional hemisphere) × 100) (Lin et al., 1993). Fluorescent immunohistochemical staining was performed with free-floating brain sections. Brain slices were washed in PBS-Triton (PBS-T) and incubated in blocking solution (1 h at room temperature; PBS-T with 4% of BSA, Sigma Life Science). Free-floating slices from ChR2- and ArchT-expressing animals were incubated in a primary antibody to GFP (chicken IgY fraction anti-GFP, 1:5000, catalog #A10262, RRID:AB_2534023, Invitrogen) in blocking solution (24-48 h at 4°C). Following repeated washes in PBS-T, sections were incubated with the secondary antibody (1:500, catalog #ab96947, RRID:AB_10681017, Abcam) in PBS-T (1 h at room temperature). Sections were then washed in PBS-T, mounted, and covered on microscope slides.

#### Axonal sprouting quantification

Four brains per experimental group were randomly chosen for axonal sprouting evaluation. Brains were fixed, frozen, and cut as previously described. Several 40 µm sections per brain were selected (approximately, from bregma 1.10 mm to bregma −0.70 mm) and stained for Vglut1, PSD-95, and DAPI. Floating sections were washed in PBS and blocked in PBS with 0.5% Triton X-100 and 10% normal donkey serum (Jackson ImmunoResearch Laboratories, code 017-000-121) (2 h at room temperature). Sections were then incubated with the following primary antibodies: chicken IgY fraction anti-GFP (ChR2^stroke^ and ArchT^stroke^, catalog #A10262, RRID:AB_2534023, Invitrogen), rabbit anti-Vglut1 (ChR2^stroke^, ArchT^stroke^, mCherry-expressing animals, 1:1000, catalog #135303, RRID:AB_887875, SYSY), goat anti-PSD-95 (ChR2^stroke^, ArchT^stroke^, mCherry-expressing animals^,^ 1:500, catalog #ab12093, RRID:AB_298846, Abcam), and mCherry, respectively (mCherry-expressing animals, 1:1000, catalog #M11217, RRID:AB_2536611, Invitrogen) in PBS containing 3% normal donkey serum and 0.5% Triton X-100 solution (overnight at 4°C). Brain slices were repeatedly washed in PBS and incubated with appropriate secondary antibodies (1:500, AlexaFluor-488 Ab96947, Abcam; all others 1:1000, Invitrogen) in PBS containing 3% normal donkey serum and 0.5% Triton X-100 solution (2 h at room temperature). A negative control (no addition of primary antibody) was conducted to confirm the antibody selectivity. Sections were further stained for DAPI (1:500 in PBS, 10 min), washed in PBS, mounted on microscope slides, and covered. Photomicrographs were acquired with Olympus Fluoview 1000-BX61 confocal microscope (Olympus, Tokyo) fitted with 60× oil-immersion objective (4× zoom, 0.5 µm step size). Three fields of interest (52.172 µm × 52.172 µm) within iS1FL and cS1FLwere imaged in three sections per animal. Imaris software (Microscopy Image Analysis Software, Bitplane, https://imaris.oxinst.com/) was used to reconstruct the 3D view of the *z* stacks and to evaluate presynaptic and postsynaptic compartments' density and volume. Briefly, background subtraction, image smoothing via Gaussian filtering, and channel intensity adjustment were applied and maintained identical for all the acquired confocal images. A preliminary stack selection was conducted to localize pucta distributed within two consecutive stacks. A puncta diameter threshold was specified at 0.6 µm and when this value was exceeded, puncta were separated on visual confirmation by the experimenter.

#### Statistical analysis

For the analyses of the 24 h recordings of stroke and sham animals, a two-level analysis was performed using linear mixed models: a first-level analysis on each animal and recording day, including temporal predictors of recording time, time since last wake epoch to estimate the homeostatic effect on individual SW characteristics across the lights on period, as well as the potential differences between the ipsilateral and contralateral hemisphere. At the second level, the parameter estimates from the first-level data for each animal for each day were used to examine the overall effects of stroke over the course of 10 d after stroke.

The potential effects of days, stroke, and stimulation group on sleep parameters and behavioral outcomes were tested using linear mixed models. Sleep and behavioral values from day 0 were assigned as a baseline predictor, while those from day 4 were used as prestimulation baseline. Main effects and interactions were tested for significance using the log-likelihood ratio test between the full model and the model without the specific factor in question. The effects between the stimulation groups were examined by *post hoc t* tests within the linear mixed model. As an exploratory analysis, macro and micro sleep parameters during the stimulation time were included as a potential predictor of behavioral outcome: percentage of NREM, number of micro-arousals, NREM-to-wake transition ratio, wave incidence, wave amplitude, wave duration, and positive and negative slope.

For the presynaptic and postsynaptic markers assessment, statistical comparisons were determined with Student's *t* test, one-way ANOVA, where corrections for multiple comparisons were conducted using Bonferroni correction, if not otherwise indicated (Prism 6 GraphPad; https://www.graphpad.com/scientific-software/prism/). Data are presented as mean ± SEM, and levels of statistical significance were set at threshold *p* < 0.05 unless otherwise indicated. Sample sizes were defined based on previous studies (Gao et al., 2008; Jego et al., 2013; Herrera et al., 2016). For each experiment, sample numbers are indicated in the corresponding figure legends. Animals that did not perform behavioral testing were excluded from the analysis as well as mice that lost EEG/EMG signals during longitudinal measurements. Data distribution was tested for normality using the Lilliefors test on the residuals from each linear mixed model calculated and found to be normally distributed. Experiments were not conducted in blinded fashion.

## Results

### Stroke alters sleep architecture and SW profile

**Figure 1.**
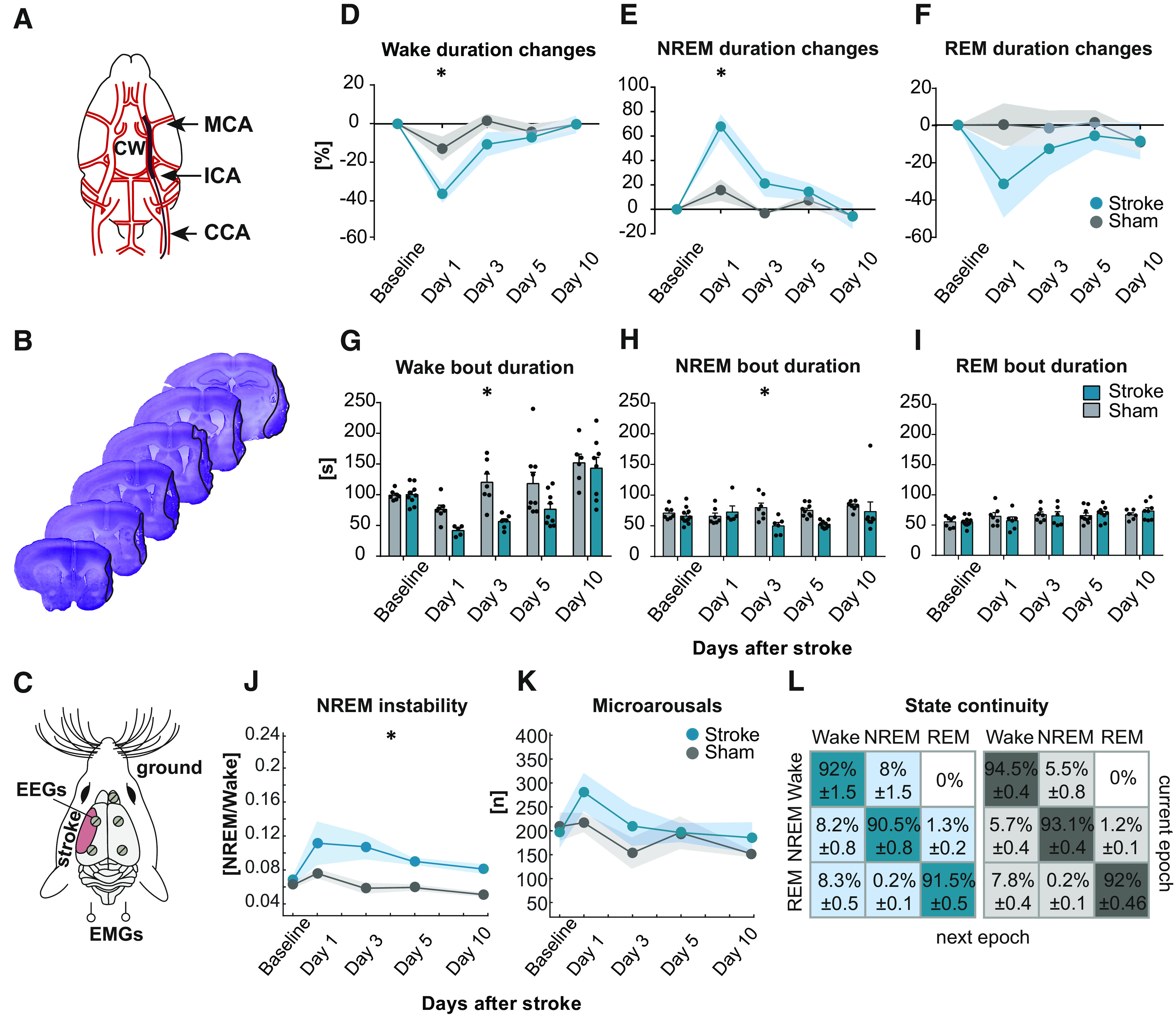
Stroke alters sleep architecture. ***A***, Schematic of the Circle of Willis (CW) with highlighted CCA, Internal Carotid Artery (ICA), and MCA, involved in MCAo procedure and filament placement. ***B***, Coronal sections (40 µm) of a representative mouse 15 d after MCAo. Nissl staining. ***C***, Schematic representation of EEG and EMG electrodes placements relative to stroke. Twenty-four hour recordings of animals' sleep-wake cycles were performed before stroke (Baseline) and again at poststroke days 1, 3, 5, and 10 in Stroke (*n* = 11) and Sham (*n* = 9) animals. ***D***, Percentage changes of wakefulness, NREM sleep (***E***), and REM sleep (***F***) total durations from each animal's baseline values. ***G***, Comparison between bout durations of wakefulness, NREM sleep (***H***), and REM sleep (***I***). ***J***, Ratio between NREM continuous episodes and transitions to wake. ***K***, Total number of microarousals in 24 h recordings. ***L***, Percentage of epochs spent in wake or sleep states for Stroke (blue table) and Sham (gray table) groups, respectively. Linear mixed model of eight matrices: Wake duration changes; NREM duration changes; REM duration changes; Wake bout duration; NREM bout duration; REM bout duration; NREM stability; and Microarousals. Data are mean ± SEM. *p < 0.0063.

SW-like oscillations are frequently observed in peri-infarct zone during NREM sleep and wakefulness (Yokoyama et al., 1996; Murri et al., 1998; Fernández-Bouzas et al., 2002). To refine the characterization of brain activity after stroke, including SW features, we first quantified the changes of sleep-wake architecture and sleep quality from animals subjected to MCAo and sham surgeries (Fig. 1*A*,*B*). Animals were chronically implanted with EEG/EMG electrodes for longitudinal sleep recordings before, and at 1, 3, 5, and 10 d after MCAo (see Materials and Methods; Fig. 1*C*). To control for multiple comparisons between the eight sleep metrics, the significance threshold was reduced to *p* < 0.0063 (i.e., 0.05/8; Bonferroni correction). MCAo resulted in an initial increase of NREM sleep duration with group differences dampening over the days recorded (Fig. 1*E*; *Day* × *Stroke* interaction: LR_(1)_ = 7.977, *p* = 0.0047). Significant main effects of stroke were found for total wake duration (Fig. 1*D*; LR_(2)_ = 22.385, *p* < 0.0001) and wake bout duration (LR_(2)_ = 34.502, *p* < 0.0001), but this general effect was not significantly different over the days after correction (*Day* × *Stroke* interaction: LR_(1)_ = 4.328, *p* = 0.0375). No significant results were observed for REM sleep total duration (Fig. 1*F*). We further explored the potential effect of stroke size within the MCAo group on all sleep architecture measures, but found no main effects or interaction effects with the day of recording (all *p* values > 0.0063).

NREM sleep instability, describing the ratio between the animals' capacity of remaining asleep compared with waking up, showed that stroke animals were significantly more likely to wake up (Fig. 1*J*; LR_(2)_ = 14.918, *p* = 0.0006).

The number of microarousals, scored as single epoch of 1 s (minimum) increased EMG signal within a NREM sleep episode, did not differ between Stroke and Sham (Fig. 1*K*; LR_(2)_ = 4.651, *p* = 0.0977).

**Figure 2.**
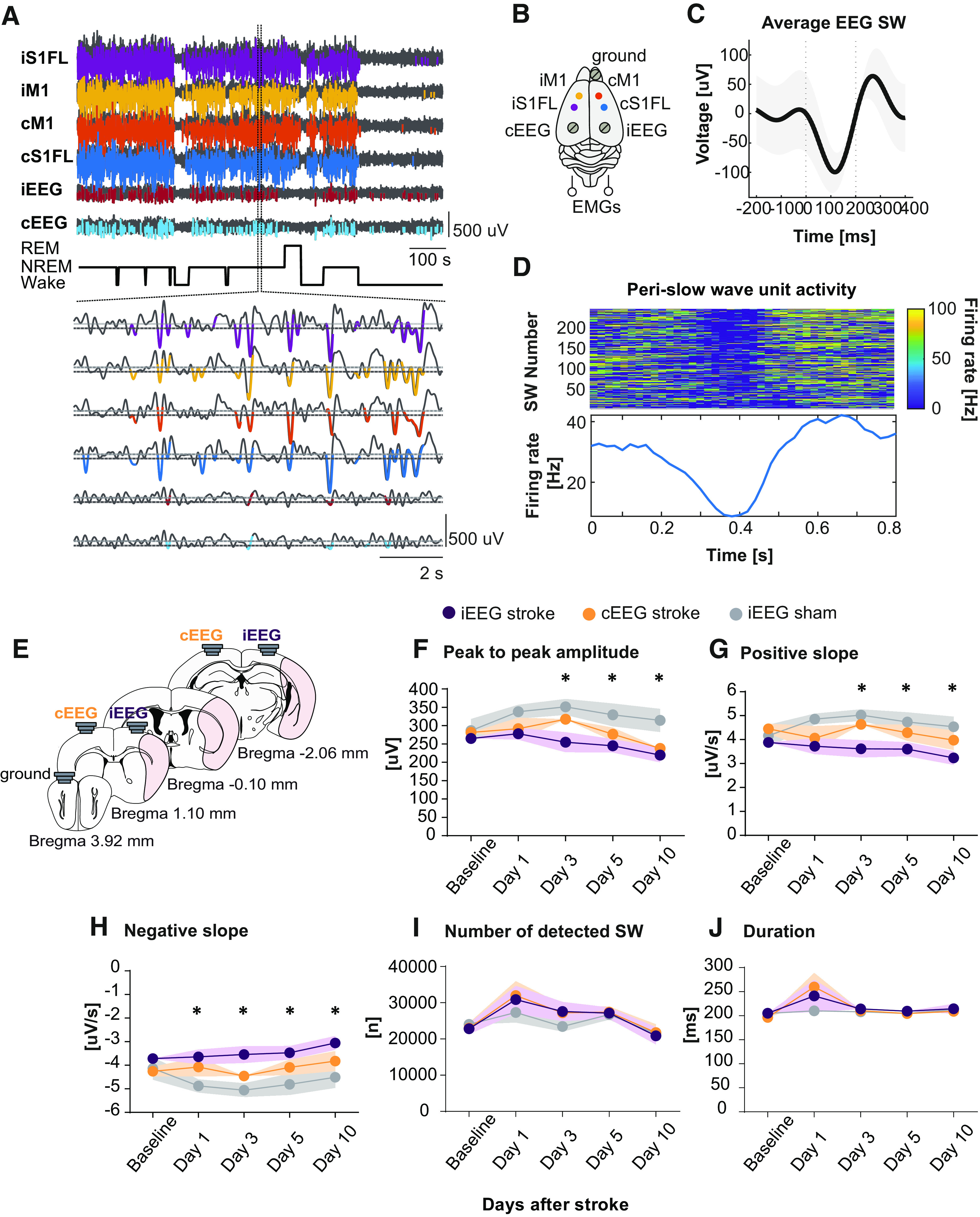
Stroke alters SW profile. ***A***, Automatic detection of single SWs from LFP recordings in iS1FL, iM1, cM1, cS1FL, and EEG traces from ipsilateral (iEEG) and contralateral (cEEG) hemispheres. Top, Representative traces in black and detected SW in colors. Bottom, Magnification of one episode of NREM sleep and detected SW. ***B***, Schematic of tetrodes and EEG/EMG electrode implantation. ***C***, Representative average SW from 24 h baseline EEG recording. ***D***, Unit activity heat map of neurons recorded during detected SW. Top, Graph represents neuronal activity suppression corresponding to the silent SW DOWN state. Bottom, Average firing rate of single units recorded during the detected SW. ***E***, Schematic of EEG electrodes position. ***F***, SW peak to peak amplitude before (Baseline) and following MCAo or sham surgery (poststroke days 1, 3, 5, and 10). ***G***, SW-positive slope. ***H***, SW-negative slope. ***I***, Number of single SWs detected. ***J***, SW duration. Stroke, *n* = 11; Sham, *n* = 9. Two-way ANOVA, followed by Bonferroni *post hoc* test. Data are mean ± SEM. *p < 0.05.

To assess SW features and changes after MCAo stroke, animals were prepared for simultaneous recordings of EEG/EMG, LFPs, or single-unit/multiunit activities in iS1FL, cS1FL, iM1, and cM1 layer V (for illustration, see Materials and Methods; Fig. 2*B*). Clear periods of neuronal quiescence corresponding to cortical DOWN states confirmed the selectivity of our SW detection method (for detection criteria, see Materials and Methods; Fig. 2*A–D*). Indeed, perilesional tetrode recordings of unit activity in S1FL showed suppression, and subsequent increase, in neuronal activity (Fig. 2*D*, top), validating the average unit firing rate observed during the detected SW (Fig. 2*D*, bottom). Both local and global SW occurred across all recorded neocortical areas (Fig. 2*A*), consistent with previous reports in rodents and humans (Huber et al., 2004; Vyazovskiy et al., 2011). If individual waves are detected across the 24 h period, we observed a significant reduction of ipsilateral SW amplitude by −13.2 ± 7.3% after stroke compared with sham controls that persisted for up to 10 d after stroke (Fig. 2*F*; day 10: −15.4 ± 6.4%; *F*_(2,70)_ = 13.82, *p* < 0.0001; two-way ANOVA, followed by Bonferroni *post hoc* test). These findings are consistent with hemispheric stroke in rodents and human subjects (Ahmed et al., 2011; Poryazova et al., 2015). Moreover, the SW-positive slope was reduced within the ipsilateral area of Stroke animals (Fig. 2*G*; *F*_(2,76)_ = 13.02, *p* < 0.0001), whereas the negative slope increased (Fig. 2*H*; *F*_(2,76)_ = 15.89, *p* < 0.0001). No significant changes were found in the number of detected SWs (Fig. 2*I*; *F*_(2,87)_ = 0.693, *p* = 0.503) or their duration (Fig. 2*J*; *F*_(82,83)_ = 0.744, *p* = 0.478; two-way ANOVA, followed by Tukey *post hoc* test). When exploring for a homeostatic effect, we did not find differences in amplitude between Stroke and Sham for ipsilateral and contralateral EEG traces (LR_(2)_ = 0.940, *p* = 0.625) nor for *time of night* (LR_(2)_ = 3.791, *p* = 0.150).

### SW^opto^ revealed a critical window of intervention after stroke

**Figure 3.**
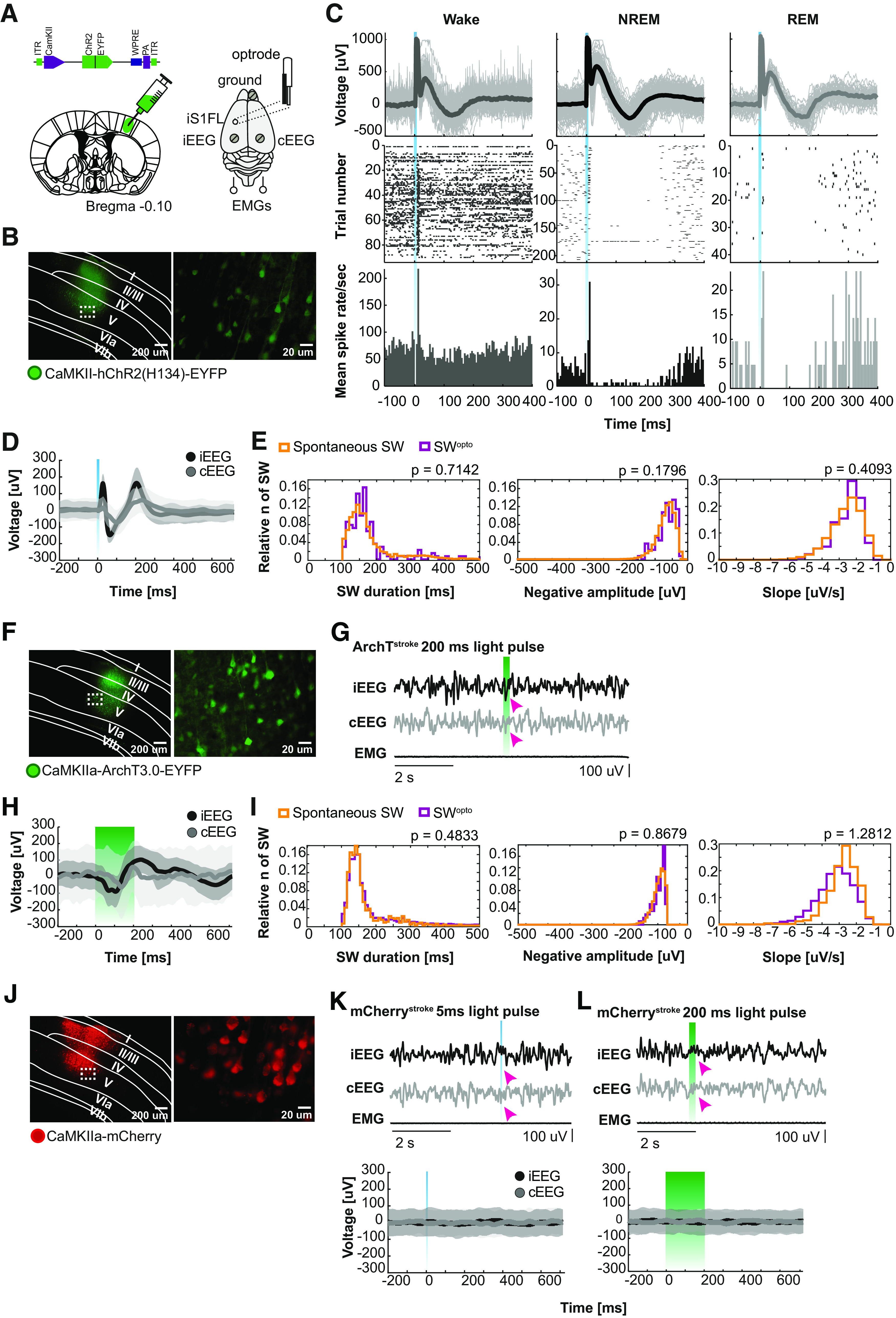
Optogenetic induction of SW-like bistable oscillations. ***A***, Scheme of a coronal brain section with AAV injection site (left), AAV structure (top), and optrode/EEG/EMG implantation representation (right). ***B***, Opsin distribution within the peri-infarct iS1FL following AAV injection of CaMKII-ChR2-EYFP. ***C***, LFP traces, single-unit activity, and correspondent raster plot and mean spike rate on optogenetic stimulation during wakefulness (left), NREM sleep (middle), and REM sleep (right) from one representative stimulation session. ***D***, Average ipsilateral (iEEG) and contralateral EEG (cEEG) traces response to activation (ChR2) of pyramidal neurons with 5 ms of single laser light pulses (473 nm). ***E***, Comparison between spontaneous and optogenetically evoked SW (SW^opto^) duration (left), negative amplitude (middle), and slope (right) during NREM sleep for ChR2-stimulated animals. Wilcoxon rank sum test, statistically significant if *p* < 0.05. ***F***, ArchT distribution within iS1FL. ***G***, Representative EEG/EMG traces on silencing of pyramidal neurons with 200 ms of single laser light pulses (532 nm) during NREM sleep. ***H***, Average iEEG and cEEG responses to ArchT stimulation. ***I***, Comparison between SW^opto^ duration (left), negative amplitude (middle), and slope (right) during NREM sleep for ArchT-stimulated animals. Wilcoxon rank sum test, statistically significant if *p* < 0.05. ***J***, mCherry (control) expression in iS1FL. ***K***, representative EEG/EMG responses during 5 ms light pulse stimulation (top) and average EEGs (bottom). ***L***, Representative EEG/EMG traces response to 200 ms pulse stimulation in one mCherry-transfected mouse (top) and its average EEGs response.

**Figure 4.**
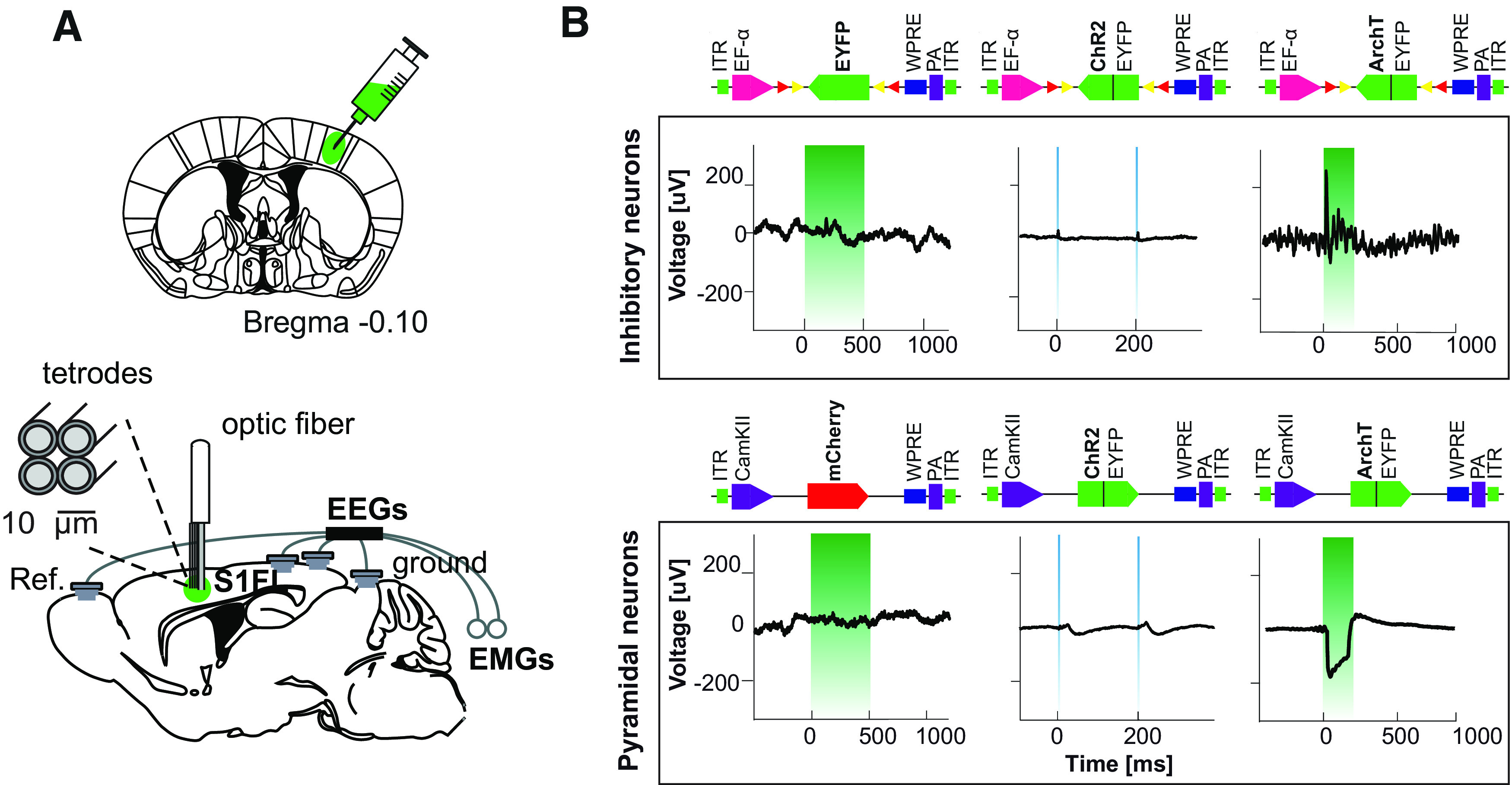
Optogenetic screening for SW induction in the peri-infarct zone. ***A***, Coronal section indicating the AAV injection site (left S1FL) (top) and schematic of EEG/EMG/optic fiber/tetrode implantation (bottom). ***B***, LFP recordings from S1FL on laser light stimulation (500 ms, 5 Hz and 200 ms) of inhibitory (top) or pyramidal neurons (bottom), respectively.

**Figure 5.**
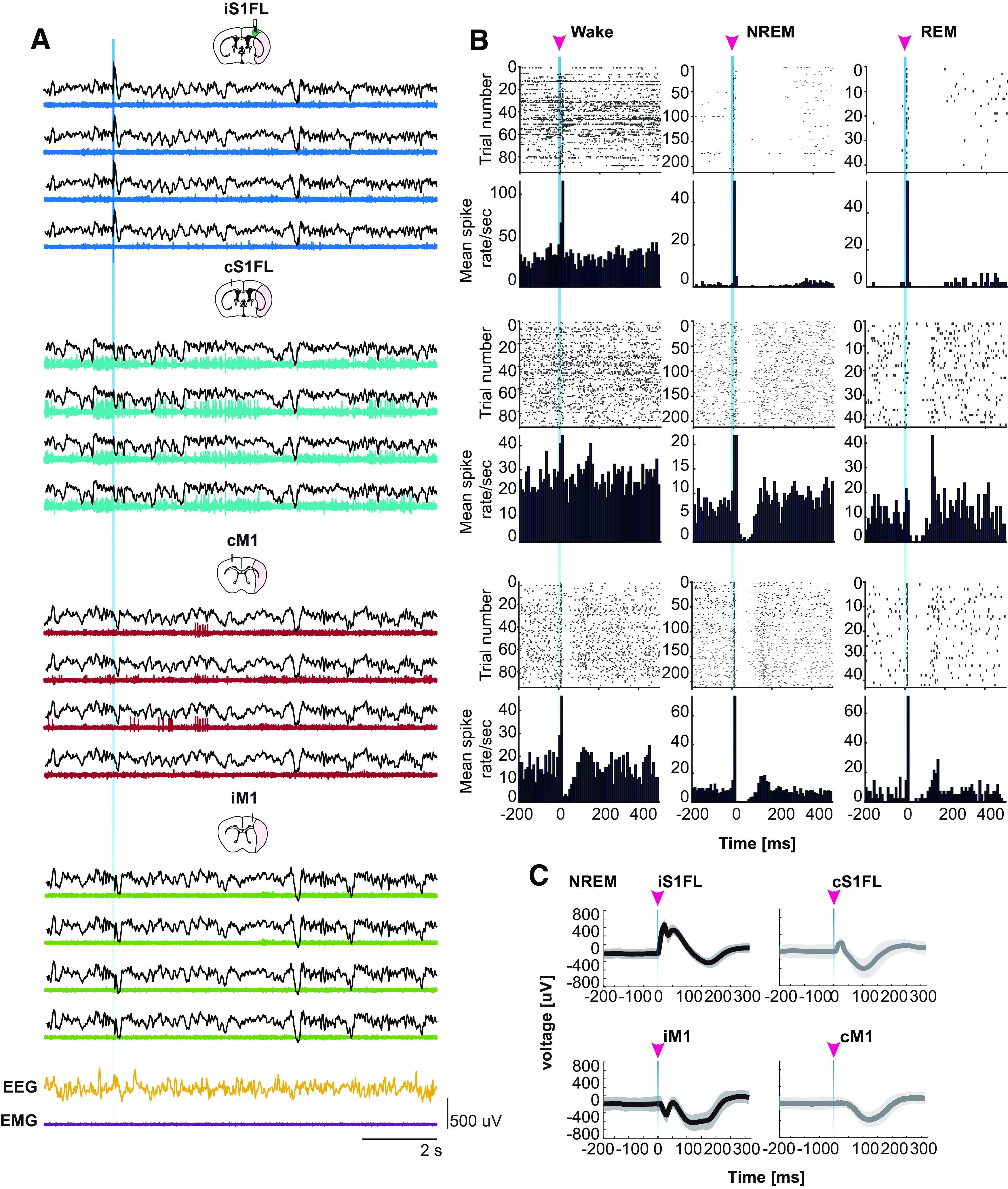
SW^opto^ oscillations travel across hemispheres. ***A***, LFP traces, multiunit activity from tetrodes placed in iS1FL, cS1FL, iM1, and cM1 and EEG/EMG traces recorded during one stimulating session showing the traveling characteristic of the evoked waves (SW^opto^). ***B***, Raster plots corresponding to one single light pulse stimulation event during wakefulness, NREM, and REM sleep as well as relative mean spike rate for iS1FL, cS1FL, and cM1, respectively. No unit was found for iM1. ***C***, Average LFP traces during the stimulation events for the four recorded cortical areas, respectively.

Here, we aimed at identifying the effect of SW^opto^ on the recovery of motor function following MCAo stroke in mice. Thus, we genetically targeted the expression of opsins to pyramidal neurons in layer V of the neocortex, given their implication in the generation of slow oscillations (Beltramo et al., 2013; McCormick et al., 2015). To achieve this, we stereotactically injected AAV2 viruses carrying ChR2, ArchT, or mCherry gene cassettes under CaMKII promoter in iS1FL (Fig. 3*A*,*B*) before animals were chronically implanted with EEG/EMG electrodes, tetrodes in cS1FL, iM1, and cM1 cortices (layer V), and a single optrode in iS1FL (see above and Materials and Methods; Fig. 3*A*). We first optimized the frequency and duration of optogenetic stimulations to mimic NREM sleep SW in both WT and *VGAT-Cre* transgenic mice to modulate excitatory or inhibitory neurons in iS1FL with 5 Hz, 1 ms light pulses (activation protocol), or 100, 200, or 500 ms single pulses (silencing protocol) (Fig. 4). We found that 5 ms optogenetic activation of iS1FL ChR2-expressing pyramidal neurons induced a short UP-like state, followed by a DOWN-like state, indistinguishable from spontaneous NREM sleep SW (Fig. 3*C–E*). Similar SW^opto^ waveform profiles were obtained on 200 ms optogenetic silencing of iS1FL ArchT-expressing pyramidal neurons (Fig. 3*F*,*I*). In the latter condition, the duration of the optogenetic silencing of iS1FL ArchT-expressing pyramidal neurons corresponded to the average duration of spontaneous NREM sleep DOWN states (Fig. 2*C*; duration: 205.2 ± 4.4 ms; Fig. 3*H*). Offline analysis confirmed that SW^opto^ duration, negative amplitude, and slope were indistinguishable from naturally occurring NREM sleep SW from the same animal (Fig. 3*E*,*I*). SW^opto^ propagated to contralateral recording sites, where SW^opto^ of variable amplitudes was recorded in EEG, LFP, and single-activity traces (Figs. 3*D*,*H*, 5). No changes in EEG features were observed in control conditions (Fig. 3*J–L*).

**Figure 6.**
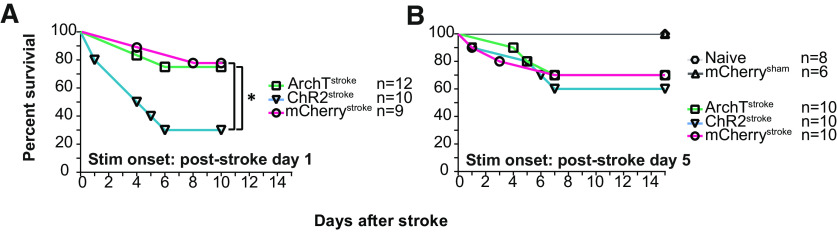
SW^opto^ defines a critical window of intervention for stroke recovery. ***A***, When the stimulation protocol started at poststroke day 1, ChR2^stroke^ animals in particular showed lower survival percentage. ***B***, When the stimulation instead began at poststroke day 5, ChR2^stroke^ animal showed an improvement in survival percentage. *p < 0.05.

To determine the optimal window for optogenetic intervention after MCAo, we evaluated the effect of this stimulation parameters on the survival rates of stroke animals. Strikingly, we observed that ChR2^stroke^ animals had lower survival rate than ArchT^stroke^, and mCherry^stroke^ mice when the optogenetic manipulation started on poststroke day 1 (single 5 or 200 ms light pulses, at 473 or 532 nm, respectively, randomly distributed over 2 h, daily; Fig. 6*A*; χ^2^_(2)_ = 7.941, *p* = 0.018; ChR2^stroke^: 30% survival; ArchT^stroke^: 75% survival; mCherry^stroke^: 77.7% survival; Log-rank Mantel-Cox test) compared with day 5 (Fig. 6*B*; χ^2^_(4)_ = 6.383, *p* = 0.172; ChR2^stroke^: 60% survival; ArchT^stroke^: 70% survival; mCherry^stroke^: 70% survival; mCherry^sham^: 100% survival; Naive: 100% survival; Log-rank Mantel-Cox test). These findings are consistent with an increased excitotoxicity after stroke (Nudo, 2006; Allman et al., 2016); hence, all our optogenetic experiments started on day 5.

### SW^opto^ during sleep improves functional recovery

**Figure 7.**
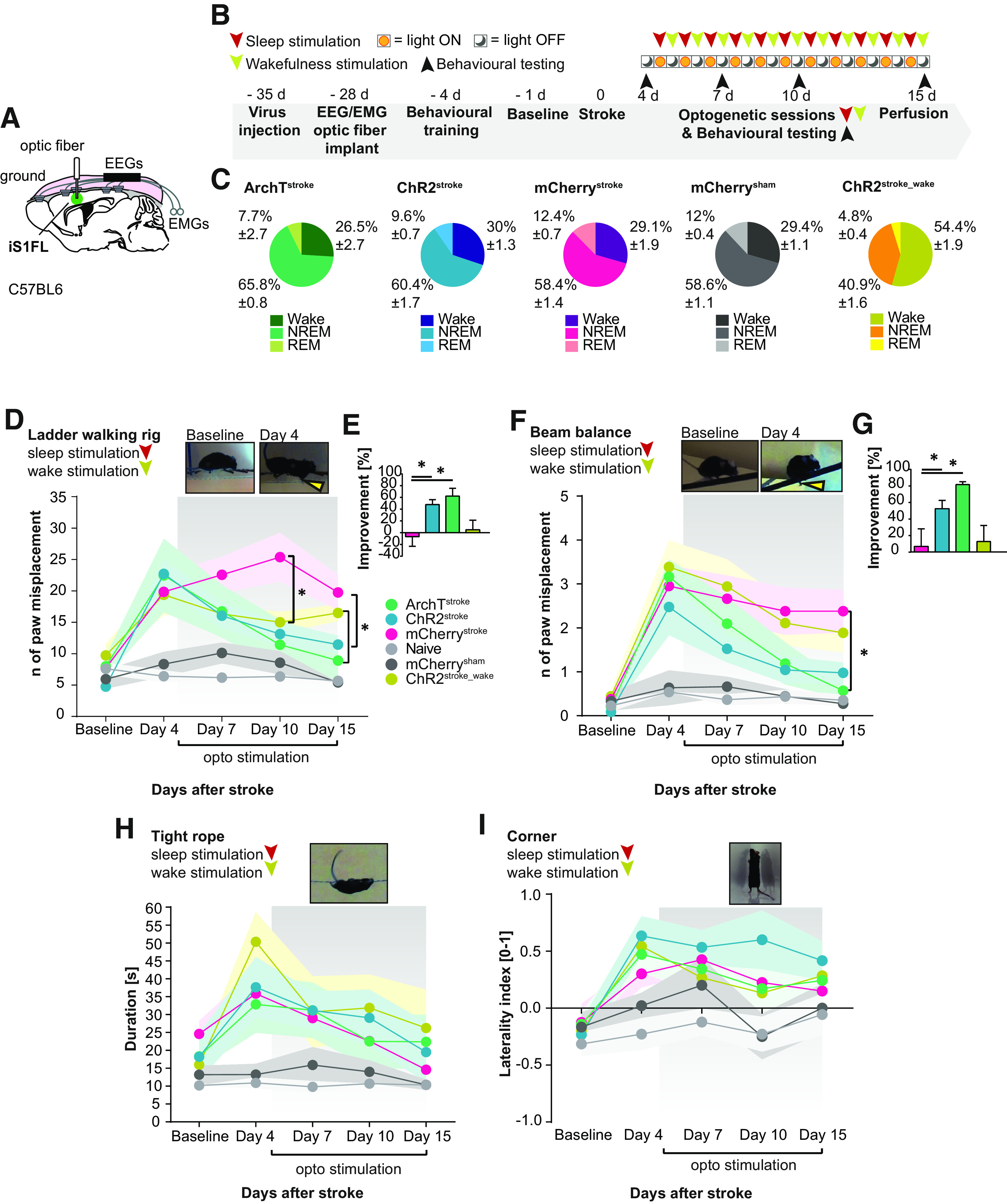
SW^opto^ during sleep improves functional recovery after stroke. ***A***, Schematics of optic fiber/EEG/EMG implantation with opsin expression site. ***B***, Experimental timeline. ***C***, Average number of single light pulses within sleep stages during the stimulation sessions of ArchT^stroke^, ChR2^stroke^, mCherry^stroke^, mCherry^sham^, and ChR2^stroke_wake^, respectively. ***D***, Stimulated animals showed better performances in the ladder walking rig test compared with mCherry-control animals (Naive *n* = 8, mCherry^sham^ *n* = 8, ArchT^stroke^ *n* = 4, ChR2^stroke^ *n* = 7, mCherry^stroke^ *n* = 6). Induction of SWs mainly during wakefulness (ChR2^stroke_wake^ *n* = 6) did not result in faster improvement of performance compared with ChR2^stroke^ stimulated during NREM sleep. Linear mixed model. ***E***, Percentage of improvement from poststroke day 4 to poststroke day 15 for ChR2^stroke^ groups and ArchT^stroke^ compared with mCherry^stroke^ control (one-way ANOVA). ***F***, Similar results were observed for performances in the balance test (Naive *n* = 8, mCherry^sham^ *n* = 6, ArchT^stroke^ *n* = 7, ChR2^stroke^ *n* = 7, mCherry^stroke^ *n* = 7, ChR2^stroke_wake^ *n* = 6). ***G***, Balance beam percentage of improvement from poststroke day 4 to poststroke day 15 for ChR2^stroke^ groups and ArchT^stroke^ compared with mCherry^stroke^ control (one-way ANOVA). ***H***, Tight rope test and corner test (***I***) did not show differences between stimulated and control groups (Naive *n* = 8, mCherry^sham^ *n* = 6, ArchT^stroke^ *n* = 7, ChR2^stroke^ *n* = 6, mCherry^stroke^ *n* = 8, ChR2^stroke_wake^ *n* = 6). *p < 0.05.

We next tested whether sleep-specific SW^opto^ improves functional recovery after MCAo in mice. The expression of ChR2, ArchT, and mCherry was genetically targeted to iS1FL pyramidal neurons as described above (Fig. 3*A*), before animals were chronically implanted with a unilateral optic fiber on iS1FL and EEG/EMG electrodes for simultaneous optogenetic control and polysomnographic recordings in freely moving mice (Fig. 7*A*; see Materials and Methods). Sparse SW^opto^ were randomly distributed during sleep starting 5 d after stroke until day 15 (single 5 or 200 ms light pulses, at 473 nm or 532 nm, respectively, randomly distributed over 2 h, daily; *n* = ∼300 optical stimuli; Fig. 7*B*,*C*).

Evaluation of the animals' fine motor movements, coordination, strength, and asymmetry at poststroke day 4 (Fig. 7*B*) showed severe behavioral deficits in all animals subjected to MCAo. Indeed, on poststroke day 4, stroke-induced animals were no longer able to finely coordinate their grasping movements (Fig. 7*D*; LR_(1)_ = 27.498, *p* < 0.0001; Fig. 7*F*; LR_(1)_ = 32.205, *p* < 0.0001). As expected, no behavioral impairments were found in mCherry^sham^ and Naive animals (Fig. 7*D* and Fig. 7*F*, respectively, *p* > 0.05).

In the ladder walking rig test (Fig. 7*D*), a significant interaction between the *stimulation group* and *days* was found (LR(5) = 11.976, *p* = 0.035). *Post hoc* analysis revealed that the ArchT^stroke^ group recovered at a faster pace than ChR2^stroke_wake^ (*t*_(101)_ = 2.842, *p* = 0.005). Generally, all mice improved across days (LR(6) = 28.235, *p* < 0.001). Main effects of stimulation group were also found (LR(10) = 42.949, *p* < 0.001). ChR2^stroke^ and ChR2^stroke_wake^ were significantly different from mCherry^stroke^ (*t*_(101)_ = −2.430, *p* = 0.017; *t*_(101)_ = −3.137, *p* = 0.002). For the beam balance (Fig. 7*F*), we found a significant interaction effect of *stimulation group* and *day* (LR(5) = 14.171, *p* = 0.015). mCherry^stroke^ did not show a significant improvement across days (*t*_(152)_ = −1.671, *p* = 0.097). However, compared with mCherry^stroke^, Archt^stroke^ mice showed significantly more improvement over the course of days (*t*_(110)_ = −2.866, *p* = 0.005). ChR2^stroke^ mice also significantly improved across days after stroke (*t*_(152)_ = −4168, *p* < 0.001), but this improvement was less than the ArchT^stroke^ group (*t*_(110)_ = −2.285, *p* = 0.024) and on par with the mCherry^stroke^ group (*t*_(110)_ = 0.580, *p* = 0.563). Comparisons of animal improvement between poststroke day 4 and 15 confirmed the functional recovery of ChR2^stroke^ (Fig. 7*E*; *t*_(14)_ = 3.46, *p* = 0.007; Fig. 7*G*; *t*_(18)_ = 2.372, *p* = 0.029) and ArchT^stroke^ (Fig. 7*E*; *t*_(14)_ = 3.083, *p* = 0.008; Fig. 7*G*; *t*_(18)_ = 3.895 *p* = 0.002; one-way ANOVA), compared with mCherry^stroke^ control. In contrast, optogenetic intervention after stroke did not lead to any improvement of motor endurance, strength (Fig. 7*H*), or asymmetry (Fig. 7*I*).

### SW^opto^ increases axonal sprouting

Stroke triggers a cascade of molecular and cellular changes, including synaptogenesis, neurogenesis, and axonal sprouting in peri-infarct zone and remote connected circuits (Nudo, 1997; Carmichael et al., 2017).

**Figure 8.**
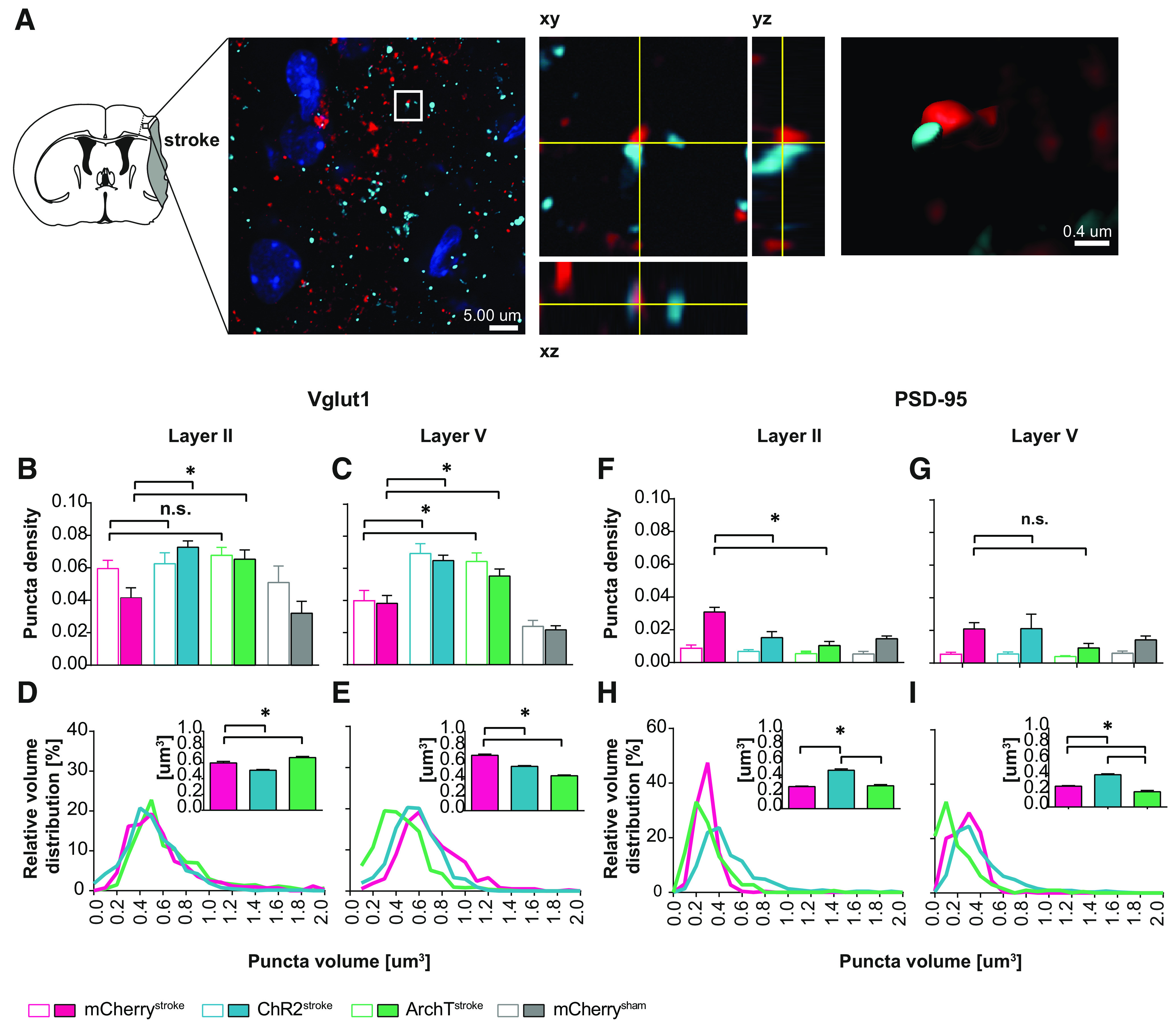
SW^opto^ increases axonal sprouting during stroke recovery. ***A***, Scheme of a brain coronal section 15 d after stroke (end point of experiment), representing tissue atrophy corresponding to the stroke area. Confocal micrography of iS1FL with 3D reconstruction of presynaptic and postsynaptic markers contact (right). Blue represents DAPI staining. Red represents Vglut1 presynaptic marker. Aquamarine represents PSD-95 postsynaptic marker. ***B***, Comparison of Vglut1 puncta density between iS1FL and cS1FL cortical areas (mCherry^stroke^ *n* = 4, ChR2^stroke^ *n* = 4, ArchT^stroke^ *n* = 4, mCherry^sham^ *n* = 4) in ipsilateral (*F*_(3,19)_ = 10.49, *p* = 0.0003) and contralateral layers II (*F*_(3,19)_ = 1.069, *p* = 0.385), as well as in ipsilateral (*F*_(3,18)_ = 16.02, *p* < 0.0001) and contralateral layers V (*F*_(3,21)_ = 11.05, *p* = 0.0001) (***C***). ***D***, Vglut1 puncta volume distribution in iS1FL layer II (*F*_(2,1630)_ = 34.85, *p* < 0.0001) and layer V (***E***) summarized in bar graph (*F*_(2,1617)_ = 155, *p* < 0.0001). ***F***, Comparison of PSD-95 puncta density in ipsilateral (*F*_(3,23)_ = 8.609, *p* = 0.0005) and contralateral (*F*_(3,21)_ = 1.105, *p* = 0.369) layers II, as well as in ipsilateral (*F*_(3,24)_ = 1.095, *p* = 0.370) and contralateral (*F*_(3,24)_ = 2.498, *p* = 0.083) layers V (***G***). ***H***, PSD-95 puncta volume distribution in iS1FL layer II summarized in bar graph (*F*_(2,2070)_ = 9.164, *p* = 0.0001). ***I***, iS1FL layer V (*F*_(2,2111)_ = 75.13, *p* < 0.0001). One way-ANOVA. Data are mean ± SEM. *p < 0.05.

To quantify the anatomic changes induced by chronic SW^opto^, we quantified the expression of presynaptic Vglut1 and postsynaptic PSD-95 proteins as a direct measurement of axonal sprouting in cortical layers V (Liu et al., 2007; Sun et al., 2017) and connected circuits in layers II (Binzegger et al., 2004; Adesnik and Naka, 2018) (Fig. 8*A*). Puncta density quantification in both iS1FL and cS1FL cortices revealed significantly higher Vglut1 protein levels in ipsilateral layer II (Fig. 8*B*; *F*_(3,19)_ = 10.49, *p* = 0.0003), and layer V (Fig. 8*C*; *F*_(3,18)_ = 16.02, *p* > 0.0001; one-way ANOVA) from ChR2^stroke^ and ArchT^stroke^ animals compared with mCherry controls. Consistently, analysis of Vglut1-positive puncta volume distribution revealed a significant increase of smaller, newly formed puncta within ipsilateral layer V of both ArchT^stroke^ and ChR2^stroke^ compared with mCherry^stroke^ animals (Fig. 8*E*; *F*_(2,2111)_ = 75.13, *p* < 0.0001). This was also true for *ex novo* Vglut1 puncta in postsynaptic sites of layer II from ChR2^stroke^ animals (Fig. 8*D*; mCherry^stroke^ vs ChR2^stroke^ *t*_(2070)_ = 4.181, *p* < 0.0001), but not ArchT^stroke^ animals (mCherry^stroke^ vs ArchT^stroke^ *t*_(2070)_ = 3.015, *p* = 0.0078, ChR2^stroke^ vs ArchT^stroke^ *t*_(2070)_ = 0.903, *p* > 0.999; one-way ANOVA, followed by Bonferroni correction).

These presynaptic changes were concomitant to a significant decrease of postsynaptic PSD-95 protein expression in iS1FL layer II of both ChR2- and ArchT-expressing animals compared with control group (Fig. 8*F*; *F*_(3,23)_ = 8.609, *p* = 0.0005; one-way ANOVA), with no differences in layer V (Fig. 8*G*; *F*_(3,24)_ = 1.095, *p* = 0.370; one-way ANOVA). PSD-95-positive puncta volume was significantly larger in iS1FL layer II (Fig. 8*H*; *F*_(2,625)_ = 85, *p* < 0.0001) and layer V from ChR2^stroke^ animals compared with mCherry^stroke^ or ArchT^stroke^ (Fig. 8*I*; *F*_(2,2111)_ = 75.13), *p* < 0.0001; one-way ANOVA).

## Discussion

Stroke is a debilitating neurological disorder, and one of the worldwide leading causes of adult disability and death in the aging population. A better understanding of the complex pathophysiological mechanisms underlying the stroke event, and the following brain plasticity warrants the improvement of existing strategies and the development of alternative therapies for stroke recovery (Feigin et al., 2017).

Here, we showed that MCAo induced an ipsilateral reduction of spontaneous SW amplitude, associated with sleep fragmentation and increased NREM sleep after stroke onset (Giubilei et al., 1992; Vock et al., 2002; Baumann et al., 2006; Hermann et al., 2008). Our results further indicate that sleep-specific optogenetic neuromodulation of brain activity after stroke had no effects on the sleep-wake cycle architecture, but it improved fine skilled motor movements compared with wakefulness interventions. These manipulations were accompanied by axonal sprouting of local and connected circuits, suggesting a direct role for SW in promoting anatomic and functional plasticity of neural circuit during sleep (Carmichael and Chesselet, 2002; Aeschbach et al., 2008; Tononi and Cirelli, 2014). Collectively, these findings emphasize a role for NREM sleep SW as a window of intervention during stroke recovery, and a possible mechanism underlying the improvement of rehabilitative strategies using repetitive transcranial magnetic stimulation (Kim et al., 2006; Brodie et al., 2014) and transcranial direct current stimulation (Boggio et al., 2006; Lindenberg et al., 2010).

Spontaneous sleep SWs are associated with neuroplastic changes (Tononi and Cirelli, 2006; Puentes-Mestril and Aton, 2017), inflammatory and immunologic adaptative response (Irwin and Cole, 2011), protective functions during infection (Irwin, 2019), and metabolic clearance (Xie et al., 2013). Clinical studies reported significant improvement in stroke rehabilitation on noninvasive brain stimulation during sleep (Niimi et al., 2019) and SW enhancement (Ebajemito et al., 2016). We used physiologically relevant stimulation protocols to avoid neuronal hypersynchrony, unnatural firing activities, and circuit adaptation by using single optogenetic stimuli randomly distributed across sleep in freely moving animals. These sparse optogenetics interventions induced SW^opto^ without perturbing sleep-wake cycle architecture. Our strategy contrasts from other studies that use long-lasting hypersynchronous optogenetic activation independently of the animal behavior, sleep-wake states, or delivered during anesthesia (Cheng et al., 2014; Lu et al., 2017; Shah et al., 2017; Tennant et al., 2017). Our findings show that sparse SW^opto^ delivered during sleep improved behavioral outcomes, whereas SW^opto^ during wakefulness did not. An explanation for this striking difference is that low-frequency, high-amplitude waves during wakefulness represent dysfunctional waves, typical of pathologic conditions that are often associated with functional abnormalities, including deafferentiated or lesioned thalamocortical circuits (Steriade et al., 1993; Butz et al., 2004). These results further emphasize the importance of sleep as a window for optimal modulation of brain activity that potentiates the effect of SW^opto^ on brain plasticity and behavioral outcomes (see below).

### Alteration of sleep-wake cycle and SW

Our findings revealed that stroke injury induces a dramatic increase in NREM sleep on the day following stroke. This effect is accompanied by transient perturbation of the circadian sleep distribution across the light/dark cycle. Although the causes of these transient changes remain unclear, they may result from a functional adaptation to the strong fragmentation of both NREM sleep and wakefulness.

Our experimental results are consistent with the sleep fragmentation, the increase in NREM sleep Stages 1 and 2, and the decreased REM sleep observed during the first days following stroke in human (Giubilei et al., 1992; Vock et al., 2002). Sleep fragmentation may result from a lack of consolidated synchrony of neuronal activity among thalamocortical circuitries, as suggested by the decreased amplitude and positive slope of spontaneous SW after stroke observed in our study. These SW profiles are indicative of low spiking synchrony of thalamic and cortical neurons (Huber et al., 2004; Vyazovskiy et al., 2009a), which may facilitate arousal on wake-promoting inputs of subcortical origins (Adamantidis et al., 2007; Carter et al., 2010; Herrera et al., 2016; Gent et al., 2018). Whether the SWs remaining after stroke are generated by a similar mechanism and support similar cortical functions, as the naturalistic SW recorded from an intact brain remains to be examined in light of the different cells types potentially implicated in SW generation (Gerashchenko et al., 2008; Cardin et al., 2009; Stroh et al., 2013; Jackson et al., 2016; Niethard et al., 2016). An important characteristic of spontaneous sleep SWs is their propagation pattern across the brain cortex, originating at anterior regions and traveling to posterior directions (Massimini et al., 2004; Gent et al., 2018). Investigating SWs' traveling changes across the ipsilateral hemisphere and the peri-infarct zone specifically represents an interesting additional aspect to explore in future work. The experimental preparation of the present study (single EEG trace per hemisphere) limited further SW analysis in this direction.

### SW^opto^ promotes behavioral recovery after stroke

Chronic SW^opto^ over 11 d after stroke facilitated spontaneous functional recovery, while earlier interventions exacerbated brain injury and decreased the survival rate of the animals, possibly because of excessive glutamate release (Lai et al., 2014), leading to increased excitotoxicity (Nudo, 2006; Allman et al., 2016). This window of spontaneous recovery is limited to a month in rodents, and 3 months in humans, during which molecular and structural changes potentiate the responsiveness to rehabilitative treatments (Murphy and Corbett, 2009; Ng et al., 2015) and emphasize a crucial intervention timeframe (Dromerick et al., 2009). Although poststroke excitotoxicity might be an accurate explanation for the detrimental effect observed in animals' survival, additional studies are required to further scrutinize markers of excitotoxicity (e.g., levels of glutamate, NMDA receptors, AMPA receptors, and their activation, caspases, ROS) in combination with optogenetic intervention at several time points following stroke.

An interesting finding in our study is that SW^opto^ had no direct effects on sleep architecture, but induced a delayed increase of sleep duration. This result is in agreement with studies showing prolonged NREM sleep on activation of somatostatin interneurons (Funk et al., 2017) and, to a lesser extent, pyramidal neurons (Rodriguez et al., 2016) in the neocortex. Noteworthy, increased NREM sleep following SW^opto^ intervention was present only within the first 2 d of stimulation (data not shown), presumably because of the brain recovery processes or the adaptation of the sleep-promoting circuits to the SW^opto^, or both. Although we cannot rule out a possible role of this transient NREM sleep increase on the sensorimotor improvement of the animals, it is unlikely that these early and transient changes are responsible for the motor improvements observed at the end of the experiment.

### SW, plasticity, and axonal sprouting

In our experiments, sensorimotor improvement after stroke was achieved either by chronic optogenetic activation, or silencing of iS1FL pyramidal neurons in freely moving mice, supporting an essential role for UP-DOWN states in brain plasticity, rather than neuronal activation or silencing alone (Puentes-Mestril and Aton, 2017). These bistable states during NREM sleep (here, mainly SW^opto^) are associated with synaptic plasticity in local circuits and their postsynaptic targets, as observed by the beneficial effect of sleep low-frequency stimulation of motor or somatosensory cortical circuits on perceptual learning (Miyamoto et al., 2016), or the formation of new dendritic spines in motor cortex (layer V) pyramidal neurons in mice (Yang et al., 2014). Furthermore, our results are in agreement with the finding that experimental disruption of cortical SW following learning impairs consolidation of visuomotor learning in humans (Landsness et al., 2009).

The early stages of stroke recovery are classically attributed to brain edema resorption and penumbra reperfusion, while later stages are associated with structural reorganization through axonal sprouting, synaptogenesis, and neurogenesis (Nudo, 2006). Here, both ChR2- and, to a lesser extent, ArchT-induced SW^opto^ promoted an increase of presynaptic and postsynaptic markers in S1FL layers V and II, respectively. Decreased PSD-95 density after SW^opto^ is consistent with similar findings on repetitive transcranial magnetic stimulation stimulation in rodents (Etiévant et al., 2015) that correlate with improved functional outcomes in nonhuman primates treated with PSD-95 inhibitors (Cook et al., 2012). Larger PSD-95 puncta were found within both layers II and V of ChR2^stroke^ animals, suggestive of a stabilization of the functional synapse (Cane et al., 2014). Thus, SW^opto^ enhance UP/DOWN state network synchronization (Gent et al., 2018), and facilitate the formation of new synapses, which are not restricted to targeted cortical circuits (i.e., pyramidal neurons in the peri-infarct zone), but also anatomically connected circuits located in ipsilateral and contralateral hemispheres (Liu et al., 2009; Cui et al., 2013). Noteworthy, brain activity in other cortical and subcortical networks, and other sleep oscillations, including spindles, participate in synaptic plasticity during NREM sleep (Rosanova and Ulrich, 2005; Chauvette et al., 2012) and may contribute to the ameliorated behavioral outcome reported here.

Collectively, our findings support a role for NREM sleep SWs in neuronal circuit plasticity and provide a clinically relevant framework for developing sparse, noninvasive neuromodulation, including acoustic brain stimulations (Ngo et al., 2013), extended transcranial magnetic stimulation or extended transcranial direct current stimulation (Ebajemito et al., 2016; Niimi et al., 2019) for optimal recovery after brain injury.
